# Using plasma biomarkers to distinguish TDP43 associated pathologies

**DOI:** 10.1002/alz.089291

**Published:** 2025-01-09

**Authors:** Joseph P Foley, Anna Zacco, Lorin Towle‐Miller, Rachel M Y Ong, Gopinath Ganji

**Affiliations:** ^1^ GSK, Collegeville, PA USA; ^2^ GSK, Stevenage, Hertfordshire United Kingdom

## Abstract

**Background:**

Mislocalization and aggregation of TAR DNA‐binding protein (TDP43) leads to loss of function and pathogenesis in 40‐45% Frontotemporal dementia (FTD) and >95% amyotrophic lateral sclerosis (ALS) patients. While TDP43 pathology has been studied in post‐mortem tissues, it has been challenging to evaluate pathology using reliable fluid biomarkers with clinical utility. We examined levels of plasma biomarkers, neurofilament light chain (NfL), glial fibrillary acidic protein (GFAP), phosphorylated Tau (pTau181) and total Tau (Tau), and followed up on previous results^1^ showing lower levels of GFAP:NfL in differentiating FTD‐TDP from FTD‐Tau subpopulations.

**Method:**

40 ALS and 32 non‐disease age‐matched control plasma samples were procured from Northeast ALS Consortium (NEALS) biorepository and PrecisionMed (Carlsbad, CA, USA). Quanterix digital immunoassays, Neuro 2‐Plex B assay, SIMOA pTau‐181 and SIMOA Tau Advantage kits, were used to determine concentrations of NfL, GFAP, pTau181 and Tau, respectively, on the SR‐X platform. Ratios, correlations and statistical analyses (unpaired t test with Welch’s correction or one‐way ANOVA with Dunnett’s test) were performed across all plasma biomarkers analyzed. Cutoffs from prior studies^1^ were examined to classify disease vs control samples. Furthermore, the ALLFTD dataset is being analyzed to evaluate relationships between some of these biomarkers and relevant clinical covariates in subgroups of interest.

**Result:**

Plasma NfL and GFAP levels were significantly (p<0.0001) elevated in ALS samples compared to non‐disease controls. NfL was elevated (∼7x) relative to GFAP (∼1.8x) in cases versus controls. Furthermore, GFAP:NFL ratio showed a notable separation with lower values being significantly (p<0.0001) associated with disease samples (mean=3.4) versus controls (mean=9.7), as previously reported^1^. Similarly, higher (∼1.8x) pTau181 and lower (∼0.7x) Tau levels were significantly (p<0.05) observed in ALS versus control samples and higher (mean=20.2) pTau181:Tau ratios achieved a markedly significant (p<0.0001) differentiation of ALS from controls. Interestingly, 80% of ALS plasma samples showed a consistent correlation of higher pTau181:Tau with lower GFAP:NFL ratios while the control samples showed the exact opposite result.

**Conclusion:**

Plasma biomarkers measured as GFAP:NfL and/or pTau181:Tau ratios have the potential to distinguish TDP43 associated pathologies such as ALS and FTD‐TDP.

^1^Cousins et al., JAMA Neurol. 2022 Nov 1;79(11):1155‐1164

